# Endorsements of five reporting guidelines for biomedical research by journals of prominent publishers

**DOI:** 10.1371/journal.pone.0299806

**Published:** 2024-02-29

**Authors:** Peiling Wang, Dietmar Wolfram, Emrie Gilbert

**Affiliations:** 1 School of Information Sciences, University of Tennessee-Knoxville, Knoxville, Tennessee, United States of America; 2 School of Information Studies, University of Wisconsin-Milwaukee, Milwaukee, Wisconsin, United States of America; University of Oviedo: Universidad de Oviedo, SPAIN

## Abstract

Biomedical research reporting guidelines provide a framework by which journal editors and the researchers who conduct studies can ensure that the reported research is both complete and transparent. With more than 16 different guidelines for the 11 major study types of medical and health research, authors need to be familiar with journal reporting standards. To assess the current endorsements of reporting guidelines for biomedical and health research, this study examined the instructions for authors (IFAs) of 559 biomedical journals by 11 prominent publishers that publish original research or systematic reviews/meta-analyses. Data from the above original sources were cleaned and restructured, and analyzed in a database and text miner. Each journal’s instructions or information for authors were examined to code if any of five prominent reporting guidelines were mentioned and what form the guideline adherence demonstration took. Seventeen journals published the reporting guidelines. Four of the five reporting guidelines listed journals as endorsers. For journals with open peer review reports, a sample of journals and peer reviews was analyzed for mention of adherence to reporting guidelines. The endorsement of research guidelines by publishers and their associated journals is inconsistent for some publishers, with only a small number of journals endorsing relevant guidelines. Based on the analysis of open peer reviews, there is evidence that some reviewers check the adherence to the endorsed reporting guidelines. Currently, there is no universal endorsement of reporting guidelines by publishers nor ways of demonstrating adherence to guidelines. Journals may not directly inform authors of their guideline endorsements, making it more difficult for authors to adhere to endorsed guidelines. Suggestions derived from the findings are provided for authors, journals, and reporting guidelines to ensure increased adequate use of endorsed reporting guidelines.

## Introduction

Reporting guidelines are structured tools for researchers to write manuscripts to report studies and findings, and for journals to ensure that the research they are publishing is both complete and transparent. Although general medical journals were born in the late 18th century, the exponential growth in scientific and medical journals did not occur until the late 20th Century [1]. To enhance the reliability and value of published health research literature, pioneers initiated the EQUATOR project in 2006, which led to the establishment of biomedical research reporting guidelines from 2010 onwards (accessible from EQUATOR (Enhancing the QUAlity and Transparency Of health Research) Network [2] cite Equator-Network) Today, many of the major reporting guidelines such as ARRIVE [3], CONSORT [4], PRISMA [5], and SPIRIT [6], have been endorsed by thousands of journals in a global scale and are accessible in different languages. Some earlier efforts on reporting guidelines were subsumed into new guidelines. For example, ASSERT (A Statement for the Scientific and Ethical Review of Trials), was subsumed into SPIRIT (Standard Protocol Items: Recommendations for Interventional Trials) [7] cite ASSERT).

The first edition of the AMA Manual of Style was published in 1962 as an in-house editorial manual, until its 7th edition in 1981, when the AMA Manual added “for Authors & Editors” to the title. The current 11th edition of the manual, published in 2020, updates the 10th edition, which was published in 2007. In the 11th edition, Chapter 2 Manuscript Preparation for Submission and Publication refers to the EQUATOR Network; Chapter 19 Study Design and Statistics, also refers to the EQUATOR Network and specific guidelines such as CONSORT, PRISMA and STROBE [8].

Although quality and transparency in scientific and scholarly communication are important in all disciplines, it is vital in biomedical and health-care fields where research findings may have life-or-death implications and impact on subsequent research. A range of reporting guidelines have been developed by various research bodies to assist researchers in the reporting of different types of research studies. According to the EQUATOR Network, a reporting guideline provides a simple, structured tool to assist in the writing of research in the form of a checklist, flowchart or structured text [2]. Reporting guidelines provide guidance and elements for authors to write research reports that are complete and meet standards for specific types of studies. Journals may recommend or require authors to adhere to the adopted reporting guidelines for submitted manuscripts.

The need for reporting guidelines to ensure research quality and completeness has been a focus of biomedical literature for years [9–11]. There have been calls requiring the use of reporting guidelines to elevate the quality of scientific reporting. For instance, an editorial [12] was simultaneously published in 28 journals to announce an initiative to mandate established reporting guidelines for all applicable submissions in the field of disability and rehabilitation.

Although hundreds of guidelines have been developed since the 1990s, challenges remain [13]. The adherence to specific guidelines has been suboptimal [14], prompting the question of whose responsibility it is to ensure that appropriate guidelines are followed when reporting research. Stakeholders include publishers, journal editors, reviewers, and the authors themselves. In a study of the online impact and dissemination of reporting guidelines, Orduña-Malea et al. [15] used link analysis based on citations to assess the impact and dissemination of CONSORT and SPIRIT guidelines, noting that the online impact of these guidelines could be improved.

Exploratory studies have investigated to what extent journals or publishers endorse specific guidelines and to what extent authors comply with endorsed guidelines. Agha et al. [16] examined 193 surgical journals to determine if the journals mentioned any reporting guidelines to authors. The majority (62%) of the journals studied for the period 2012–13 made no mention of reporting guidelines. Of the 73 journals that did mention reporting guidelines, only 10 (14%) required that reporting guidelines be used. The study authors concluded that the mention of reporting guidelines needed improvement and recommended that journals require adherence to supported guidelines. Sims et al. [17] found slightly better performance for emergency medicine journals, where 16 of the 27 journals studied mentioned at least one relevant guideline within their instructions to authors. Agha et al. [18] studied whether mandating compliance with selected reporting guidelines (STROBE, CONSORT, PRISMA) improved reporting quality. Over the three-year period of the study, compliance increased for all three reporting guidelines. However, even with author prompting, Shanahan et al. [19] found that a majority of authors involved in their study could not correctly identify relevant guidelines. More recently, Holmer et al. [20] also found that for a sample of systematic review preprints uploaded to MedRxiv, more than half of the resulting journal publications did not adhere adequately to the PRISMA 2020 checklist.

Another purpose for studying adherence to reporting guidelines is to determine if the guidelines enhance the completeness and quality of reports. Among the earliest, Smidt et al. [21] examined whether the publication of the Standards for the Reporting of Diagnostic Accuracy Studies (STARD statement) improved the quality of reporting for diagnostic accuracy studies. The authors noted that the quality had improved somewhat, with the effect most noticeable in journals that adopted the STARD statement and encouraged editors to require the mention of the STARD statement. Hopewell et al. [22] investigated the impact of the CONSORT for Abstracts guidelines on the reporting quality of abstracts of randomized trials for five general medical journals from 2006–09. The study found that the mean number of items reported per abstract increased over time, indicating that the implementation of CONSORT guidelines for abstracts improved the reporting quality of abstracts for randomized trials. Stevens et al. [23] conducted a systematic review to assess whether the completeness of reporting of health research is associated with journals’ endorsement of reporting guidelines. Nine reporting guidelines were assessed. Outcomes regarding the relationship between journals’ endorsement of reporting guidelines and the completeness of the reports were inconclusive. Similarly, Turner et al. [24] studied the impact of the endorsement of the CONSORT statement on the completeness of the reporting of randomized trials, finding that journal endorsement may benefit the completeness of the reporting of Randomized Control Trials (RCTs) but that the completeness of reporting was still sub-optimal. This is a sentiment echoed by O’Leary and Crawford [25] for biomedical journals. Blanco et al. [26] conducted a scoping review to identify, analyze and classify interventions to improve adherence to reporting guidelines. The authors identified five categories of interventions and three types of evaluative interventions that were lacking, and encouraged stakeholders to consider implementing and evaluating the identified interventions. The authors found that biomedical journal editors generally believed that engaging trained professionals would be the most effective editorial intervention, although it would be resource-intensive, and that peer reviewers should not be asked to check reporting guidelines. More recently, Speich et al. [27] conducted a similar study of SPIRIT guidelines for interventional randomized control trials. The authors reported that from 2012 to 2016, adherence to SPIRIT guidelines improved but remained modest.

In a survey, Malički et al. [28] report the responses from authors, reviewers, and editors; they found that 62% editors and 67% reviewers strongly agreed with the statement: “Journals must check and enforce appropriate reporting guidelines (e.g., those from www.equator-network.org) for disclosing key aspects of the research and data analysis.” (p. 5/13) The EQUATOR-Network recommends both editorial staff and peer reviewers be involved in checking the adherence to required reporting guidelines as the enforcement routes.

Another focus of reporting guidelines research has been to determine if the guidelines influence the peer review of reports by providing a framework by which reviewers may assess the merits of the research. Not all reviewers will bring the same level of background or experience in approaching the review process. Reporting guidelines could provide valuable guidance to peer reviewers. Cobo et al. [29] in a study of biomedical journal research reporting that included the consideration of the use of reporting guidelines by reviewers, found that there was no statistically significant positive effect by suggesting reviewers use reporting guidelines. However, they found that there was a positive effect in improving manuscript quality by adding a statistical reviewer. Cobo et al. [30] investigated the effect of providing additional reviews based on reporting guidelines such as STROBE and CONSORT on the quality of manuscripts. Similar to the previous study, reviews based on reporting guidelines did improve manuscript quality, although the observed effect was smaller than the authors hypothesized and not definitive. Vilaró et al. [31] concluded that adherence to reporting guidelines increased the number of citations a publication receives, although they believed the outcome could be an effect of the additional editorial process provided by a senior methodologist who could identify missing reporting guidelines.

Hirst and Altmann [32] examined the prevalence of journals’ instructions to peer reviewers to determine if and how reviewers are encouraged to use reporting guidelines. Fewer than half of the journals studied provided online instructions to reviewers. The authors made several recommendations, including wider reference to the EQUATOR Network online library. In a recent study, researchers in collaboration with journals asked peer reviewers to check whether specific reporting guideline items were adequately reported. One trial was about CONSORT-PR and another trial was about SPIRIT-PR. The results from 421 randomly selected manuscripts showed that reminding peer reviewers of the specific reporting items did not result in any improvement in reporting quality [33]. Although not directly focusing on reporting guidelines, Song et al. [34] conducted a scoping review of peer review guidelines to assist reviewers in the review process of research reports that included guidelines found on the EQUATOR Network. They concluded that peer review guidelines are not uniform and recommend that stakeholders must come together to standardize guidelines or recommendations for peer reviewers. Wang and Wolfram [35] examined 55 journals that published one or more guidelines to determine if instructions for the guidelines were provided to authors and reviewers. Forty of the 55 journals (72.7%) provided instructions to authors for one or more of the 11 guidelines studied. Very few journals provided instructions to reviewers regarding reporting guidelines, ranging from 0 to 8 journals for the 11 guidelines.

## Reporting guideline characteristics and endorsement

The main page of the EQUATOR Network lists 11 main study types and their reporting guidelines. The site has links to 606 reporting guidelines as of January 2024. For each study type, a reporting guideline may have several documents. For example, CONSORT provides several document types: Statement, Checklist, Flow Diagram, Abstract, and Explanation & Elaboration. These documents are published in journals or on the guideline’s website. In this study, we focused on journal endorsements for five reporting guidelines (Table 1).

**Table 1:** Observed major biomedical study types and their reporting guidelines.

Study Type	Reporting Guideline	Type of Documents	Web-Endorsements
Randomized trials	CONSORT 2010 (Consolidated Standards of Reporting Trials)	Statement; Checklist; Flow Diagram; Abstract	https://web.archive.org/web/20230203080815/http://www.consort-statement.org/about-consort/endorsers1
Trial protocols	SPIRIT 2013 (Standard Protocol Items: Recommendations for Interventional Trials)	Statement; Checklist; Figure	https://www.spirit-statement.org/about-spirit/spirit-endorsement/
Systematic reviews	PRISMA 2020 (Preferred Reporting Items for Systematic Reviews and Meta-Analyses)	Statement; Checklist; Flow Diagram	http://www.prisma-statement.org/Endorsement/PRISMAEndorsers
Case reports	CARE (CAse REports) (updated 2013)	Statement; Checklist	https://www.care-statement.org/endorsements
Animal pre-clinical studies	ARRIVE 2.0 (Animal Research: Reporting of *In Vivo* Experiments) (updated 2020)	Guidelines; Essential 10; Recommended set	https://arriveguidelines.org/supporters/journals

In describing how journals support specific guidelines, there is no agreement on distinctions between terms such as “adoption” and “endorsement” of reporting guidelines. Some studies have used the terms interchangeably [36–40]. Stevens et al. [23, p 3 of 29] provide distinctions between the related terms endorsement, adherence and implementation:

*Endorsement*—Action taken by a journal to indicate its support for the use of one or more reporting guideline(s) by authors submitting research reports for consideration; typically achieved in a statement in a journal’s “Instructions to authors.”

*Adherence*—Action taken by an author to ensure that a manuscript is compliant with items (that is, reports all suggested items) recommended by the appropriate/relevant reporting guideline.

*Implementation*—Action taken by journals to ensure that authors adhere to an endorsed reporting guideline and that published manuscripts are completely reported.

The EQUATOR Network also addresses *implementation* by three routes: “1) Endorse reporting guidelines; 2) Endorse reporting guidelines and request a checklist; 3) Require reporting guidelines, proved with a submitted checklist.” It also defines the *enforcement* routes as: “1) Editorial staff confirm checklist use; 2) Peer reviewers confirm checklist use.” [2,…/toolkits/using-guidelines-in-journals/four-possible-implementation-routes/]. For the endorsement of a reporting guideline, PRISMA states “PRISMA endorsement by journals is typically exemplified by a statement in the journal’s Instructions to Authors indicating the journal’s support of PRISMA, typically in the form of a recommendation to authors to adhere to the PRISMA checklist and to use a PRISMA flow diagram in their systematic review manuscripts.” [5] It further suggests the text for such a statement as well as “Fill out the form …… we will add you to the endorsers list.” [5].

Reporting guidelines typically list the endorsers on their websites (Table 1); however, the best and most useful way for a journal to endorse a guideline is to identify which specific reporting guideline authors should use in the instructions for authors (IFA) under the study types the journal publishes. For example,

*“Nutrition Journal* requires the submission of a populated SPIRIT checklist and SPIRIT figure for all study protocols. The figure should be included in the main body of the text and the checklist should be provided as an additional file, both the figure and the checklist should be referenced in the text.” [41].

BMC Veterinary Research, in its Preparing Your Manuscript/Research instructions, states: “Manuscripts reporting results of an animal clinical trial must conform to ARRIVE 2.0 standards, and authors should submit a completed ARRIVE checklist alongside their manuscript. In particular, authors should acknowledge the ARRIVE 2.0 Essential 10 list …… Research articles may also report on systematic reviews of published research provided they adhere to the appropriate reporting guidelines which are detailed in our editorial policies.” [42] Following BMC’s top-level link for all journals, authors will see the nine reporting guidelines listed, among which, both ARRIVE and PRISMA (for systematic reviews) are listed.

For the purpose of this study, we need to differentiate the two types of endorsements: 1) *web*-*endorsed* if the journal is listed by the reporting guideline’s website as an endorser (Table 1, Column 4), and; 2) *IFA-mentioned* if the journal suggests or requires the reporting guidelines in the IFA (as the examples above).

In a series of studies of the endorsement of CONSORT [43–45], researchers found an increase in endorsement of the guideline over three time periods: 22% in 2003, 36% in 2007 and 43% in 2014. The researchers acknowledged the increased endorsement of CONSORT but were 4concerned with the inconsistent use of endorsements across journals and publishers.

Journals have been endorsing and publishing reporting guidelines; however, studies show that it is unclear to what extent the *implementation* and *enforcement* of reporting guidelines have taken place for the most developed guidelines. The improvement of adherence to the endorsed guidelines still lacks effective strategies and methods.

The purpose of this study is to investigate to what extent journals from 11 prominent publishers of biomedical research have endorsed the five commonly used guidelines (i.e., are listed on four guidelines’ websites and/or mentioned in their IFAs). As proposed by the EQUATOR Network, editorial processes to *enforce* the endorsed guideline can involve checking by both staff and peer reviewers. Therefore, we include open peer-review journals in our data collection to observe if peer reviewers play a role in enforcing reporting guidelines.

More specifically, this study aims to investigate journal endorsements of five reporting guidelines (ARRIVE, CARE, CONSORT, PRISMA, and SPIRIT) to address the following research questions:

Q1. Which publishers and journals publish reporting guidelines?
Q2. Which journals are listed as endorsers on the websites of the reporting guidelines?
Q3. Which journals instruct authors about the reporting guidelines?
Q4. Do journals that publish reporting guidelines also instruct authors in their IFAs to adhere to the published guidelines?
Q5. Do journals that appear on guidelines’ endorsement lists also instruct authors in IFAs to adhere to the web-endorsed guidelines?
Q6. Do peer reviewers comment on reporting guidelines in their reviews?

## Materials and methods

Data were collected from five sources:

1. EQUATOR Network website: This site lists the reporting guidelines and links to the guideline’s websites, and the journals that published these guidelines. In addition, the site also provides toolkits for using guidelines.
2. Reporting guidelines’ websites: Typically, a reporting guideline has a website that publishes the guideline and its related documents. In addition, the website also invites endorsement and lists the endorsing journals.
3. Publishers’ websites: Most studies of the endorsements of individual reporting guidelines by journals have used Journal Impact Factor (JIF) as a criterion for journal selection (e.g., [33–35]). In this study, we collected data from prominent publishers’ journals and used multiple criteria to select journals for inclusion.
4. Journal websites: Instructions/information for Authors (IFA) for each journal in this study were reviewed to collect data about mentions of reporting guidelines for the five research types: trials, protocols, systematic reviews/meta-analyses, case report, and studies involving animals.
5. Articles’ Peer Reviews from open peer review (OPR) journals: Not all articles published peer reviews alongside the article. If peer review reports are available, the article’s main page will have links to the reports. Review reports for selected journals and articles were downloaded manually.

### Data collection—Journals

The data were collected and cleaned between November 1, 2022, and February 28, 2023, and were updated between November 28 and December 30, 2023. The publishers were selected based on Research.com: (1) the Best Biology and Biochemistry Journals and (2) the Best Medicine Journals. From these lists, the publishers whose journals are in the top 100 or that are specialized in biomedical areas are included; university publishers or relatively new multidisciplinary publishers were not included. Springer was treated as three publishers in this study due to Springer, Nature, and BMC having separate platforms although they are all part of Springer Nature.

The compiled dataset (*dataset-o*) included 559 biomedical journals from 11 prominent publishers (Table 2). The overarching selection criteria for journal inclusion were that the journal publishes English language original research, systematic reviews/meta-analyses, case reports, study protocols, or research involving animals. Additional criteria for inclusion were if the journals published one or more of the studied reporting guidelines, if the journal was a named flagship by the publisher (e.g., BMC Biology), or if innovative peer-review practices were adopted by the journals (e.g., open peer review). In addition, to collect a manageable number of journals from big publishers, we used the publisher’s searching, browsing, or filtering functions to narrow down the results. Because these functions and classification schemes differ among the publishers, not all criteria were applicable to all publishers. We adjusted our strategies accordingly. As an example, BMC tags their journals if the journal has adopted open or transparent peer reviews (OPR or TPR). We collected all OPR or TPR journals under the four subject categories: Biomedicine, Dentistry, Life Sciences, and Medicine & Public Health which are included in our dataset. BMJ has a Premier collection of 34 research journals for health science libraries. We included all the journals in the Premier Collection, the flagship journal (The BMJ), and six open-access journals. Elsevier’s subject access is available by selecting a Subject Area that can be narrowed down to one specialty at a time. We browsed two Subject Areas: Medicine and Biochemistry, Genetics & Molecular Biology; Elsevier’s browsing mode can filter out journals no longer in publication (i.e., journals transferred to another publisher or that have ceased publication). Taylor & Francis allows one to filter journals by ISSN (International Standard Serial Number) or no ISSN, thus we first eliminated those journals without an ISSN during the process. The smaller publishers specializing in biomedical journals include Cell Press, JAMA, The Lancet Group, and PLOS; all their biomedical journals meeting the overarching selection criteria were included.

**Table 2:** Journals published reporting guidelines.

Journal	Publisher	ARRIVE	CARE	CONSORT	PRISMA	SPIRIT
BMC Medicine	BMC			2010		
BMC Veterinary Research	BMC	2020				
BMJ	BMJ			2010	2021	
BMJ Case Reports	BMJ		2013			
Experimental Physiology	Wiley	2020				
Headache	Wiley		2013			
Int J Surg	Elsevier				2021	
JAMA	JAMA Network					2018
J of Clinical Epidemiology	Elsevier		2014	2010	2021	
J of Dietary Supplements	Taylor & Francis		2013			
J of Medical Case Reports	BMC		2013			
The Lancet	The Lancet Group			2010		2013
PLOS Biology	PLOS	2020				
PLOS Medicine	PLOS			2010	2021	
System Reviews	BMC				2021	2012
Trial	BMC			2010		2012
Veterinary Clinical Pathology	Wiley	2020				
Total	8 publishers	4	5	6	5	4

* Cell Press, Nature Research, and Springer in our datasets did not publish any of the five guidelines

### Data collection—Guidelines mentioned in journal IFAs

Each journal’s IFA was examined to code if a reporting guideline was mentioned for the type of study. For the mentioned reporting guidelines, we also recorded which document type were referenced. (See Table 1 for specific documents by the guideline). If the journal linked to the page for the reporting guidelines, we coded the report guidelines as mentioned (such as the example of BMC Veterinary Research’s link to editorial policies above). If the IFA referred to the publisher’s Author Service or Equator Network, we excluded such mention because it does not reference specific reporting guidelines. The dataset examining IFAs was stored in an MS Excel spreadsheet and was imported to an MS Access database for analysis.

### Data collection—web-endorsed journals

To examine if the 559 journals in our dataset (*dataset-o*) are listed as endorsers in reporting guidelines’ websites, we were able to scrape endorsement lists from the CONSORT, PRISMA, SPIRIT, and ARRIVE websites. (Note: The CONSORT website was under maintenance at the time of this writing. At the Wayback Machine, the archived page for our data collection period is still available as of January 10, 2024). CARE has an Endorsement page; however, besides acknowledgement that multiple journals and medical publishers or organizations have endorsed CARE, the site does not list all endorsing journals [46, Endorsements]. The endorsing journal data from the four reporting guidelines include only the titles with a URL, which can be parsed to identify a publisher, but we could not verify the accuracy of these URLs. For example, one of the ARRIVE endorsers, Vaccine Reports, has the URL “www.elsevier.com/journals/vaccine-reports/2405-7843/guide-for-authors” that resulted in a blank page. Another endorser, Acupuncture in Medicine, has the URL as a BMJ journal “aim.bmj.com/site/about/guidelines.xhtml” that will redirect readers to the journal’s new publisher, Sage Publishing.

After we imported the scraped titles with URLs into the MS Access database, we resolved title variants for some journals that were listed differently on the different endorsers lists, or typos (e.g., Electronic Journal of Biotechnology, listed as an ARRIVE endorser, has the first word misspelled; BMC Complimentary and Alternative Medicine listed as a SPIRIT endorser has the second word misspelled but this BMC journal also endorsed other three guidelines.). This dataset (*dataset-l*) includes 1,580 endorsing journals that endorsed one to four guidelines (Mean: 1.24; SD: 0.6481). Due to the lack of endorsing journals for CARE, the outcomes for research question 5 included only four reporting guidelines. Due to the lack of publishers for the web-endorsed journals, the analyses were limited to the overlap of *dataset-o* and *dataset-l*, resulting in 177 journals (*dataset-l&o*) to observe if these web-endorsed journals’ IFAs also instructed authors about the reporting guidelines.

### Data collection—peer review reports

We collected peer review reports from five journals: PLOS Biology (TPR), PLOS Medicine (TPR), BMC Medicine (OPR), Journal of Medical Case Reports (OPR), and Trials (OPR). These published peer review reports or decision letters published alongside recent articles were examined to determine if the reviewers mentioned the manuscripts’ adherence to the endorsed reporting guidelines. This dataset includes:

- BMC Medicine: 331 reviewer reports for all 115 articles published in 2020 and 2021
- Trials (BMC): 728 reviewer reports for all 399 articles published in 2020, 2021 and 2022
- PLOS Biology: 253 reviewer reports for all 83 articles published in 2021 and 2022
- PLOS Medicine: 326 reviewer reports for all 74 articles published in 2021 and 2022
- Journal of Medical Case Reports (BMC): 225 reviewer reports for all 95 articles published in 2021 and 2022

#### Data analysis

Frequency distributions for guideline mentions were tallied and inspected for the dataset containing the web-endorsed journals (*dataset-l* = 1,580), which includes all journals from the websites of the four reporting guidelines that list endorsing journals. The journals we observed for IFAs (*dataset-o* n = 559) were analyzed to identify IFA-mentions: if the journals mentioned the reporting guidelines and/or specific documents of the guidelines. The journals in both datasets above (*dataset-l&o =* 177) were analyzed to observe the two types of endorsements.

For each of the four reporting guidelines, the number of web-endorsed journals and the percentage of these journals as IFA-mentioned journals were calculated. For the IFA-mentioned journals, the specific documents recommended or required were aggregated. For journals that published one or more reporting guidelines, we focused on these journals’ IFAs. QDA Miner was used to analyze peer review reports for cases of the guideline’s enforcement in the editorial process.

## Results

This section is arranged according to the research questions.

### Journals that published reporting guidelines (Q1)

For the journals that published reporting guidelines, we examined: (1) which guidelines the journal published and, (2) if the journal mentioned the published guideline in its IFA. In Table 2, the five reporting guidelines were published by 17 journals (by BMC, 5; Wiley, 3; PLOS, 2; Elsevier, 2; BMJ, 2; JAMA, The Lancet, Taylor Francis, 1 for each).

#### Reporting guidelines web-endorsed journals (Q2)

As described in the Introduction, journals that endorse a reporting guideline may be listed on the guideline’s website as endorsers. Four reporting guidelines have listed endorsers on their websites: ARRIVE has 1047 endorsing journals, CONSORT has 575 endorsing journals, PRISMA has 190 endorsing journals; SPIRIT has 152 endorsing journals. The most endorsed reporting guideline is ARRIVE for reporting animal research, which was originally published in 2010 (current version 2.0). CONSORT, for reporting randomized trials, is the next most endorsed reporting guideline. It is endorsed by over 50% of the core medical journals listed in the Abridged Index Medicus. The guideline was also published in 2010 and subsequently with many specialized guidelines or extensions. Because the CONSORT website was unavailable at the time of the writing of this work, we relied on the web archive version of CONSORT (accurate on January 6, 2024). The four lists from the reporting guidelines’ websites were merged. The data were combined to form a uniform dataset of 1580 journals, of which 1332 (84.3%) endorsed one guideline, 151 (9.6%) endorsed two, 53 (3.4%) endorsed three, and 44 (2.8%) endorsed all four guidelines. None of the websites provided data about the journals’ publishers and many of the links from the titles resulted in 404 error messages. It is beyond the scope of this study to identify each journal’s publisher given that journals also change publishers from time to time.

Of the 559 journals published by the 11 publishers in our dataset, we identified 177 journals that web-endorsed one to four of the reporting guidelines: 109 (61.6%) journals endorsed one guideline, 28 (15.8%) journals endorsed two, 13 (7.3%) endorsed three, and 27 (15.3%) endorsed all four reporting guidelines.

Table 3 also shows that four publishers in our dataset (BMC, Cell Press, JAMA, and PLOS) have a higher percentage of their journals endorsing these four reporting guidelines. For example, all of JAMA’s journals except for one endorsed SPIRIT.

**Table 3:** Journals listed as endorsers on reporting guidelines’ websites (dataset-l).

Publisher	Observed Journals	Number of (%) Journals on Website as Endorsers				
		ARRIVE	CONSORT	PRISMA	SPIRIT	≥ One Guideline
BMC	64	41	36	40	34	50
		64.1%	56.3%	62.5%	53.1%	71.8%
BMJ	42	3	11	3	2	12
		7.1%	26.2%	7.1%	4.8%	28.6%
Cell Press	28	15				15
		53.6%				53.6%
Elsevier	70	24	13	1		28
		34.3%	18.6%	1.4%		40.0%
JAMA Network	13	1	5		12	12
		7.7%	38.5%		92.3%	92.3%
The Lancet Group	24	1	4	1	1	5
		4.2%	16.7%	4.2%	4.2%	20.8%
Nature Research	63	21	2			22
		33.3%	3.2%			34.9%
PLOS	9	7	3	2	1	7
		77.8%	33.3%	22.2%	11.1%	77.8%
Springer	59	2	1			3
		3.4%	1.7%			5.1%
Taylor & Francis	96	1	6			7
		1.0%	6.3%			7.3%
Wiley	91	8	9		1	16
		8.8%	9.9%		1.1%	17.6%
Total	559	124	90	47	51	177
		22.2%	16.1%	8.4%	9.1%	31.7%

Note: CARE does not list endorsing journals on its website

#### Journal IFA-mentions of guidelines (Q3)

Because manuscripts are submitted to specific journals, authors follow the instructions provided by those journals. Reviewing the IFAs of the 559 journals (*dataset-o*), we found that 410 journals (73.4%) mentioned at least one of the reporting guidelines and 149 journals (26.7%) did not mention any reporting guidelines. For the journals mentioning the reporting guidelines in their IFAs, 124 (30.2%) mentioned one guideline, 98 (23.9%) mentioned two, 93 (22.7%) mentioned three, 43 (10.5%) mentioned four, and 52 (12.7%) mentioned five.

The journals by the 11 publishers mentioning specific reporting guidelines are summarized in Table 4. Cell Press and PLOS all mentioned ARRIVE. Journals by JAMA, The Lancet Group, and PLOS all mentioned CONSORT and PRISMA. Journals by JAMA and The Lancet Group all mentioned SPIRIT. A majority (84.7%) of Springer’s journals mentioned CARE. Springer’s journals show a higher percentage in IFAs mentioning all five guidelines. Because not all journals publish the five study types the guidelines represent, not all journals will endorse the five reporting guidelines. Therefore, we further analyzed the set of 177 journals in our dataset that are also listed as endorsers on the four guidelines’ websites.

**Table 4:** Reporting guidelines mentioned in journal IFAs.

Publisher	Journals	Number (%) of Journals Mentioned Guidelines in IFAs					
		ARRIVE	CARE	CONSORT	PRISMA	SPIRIT	≥ One Guideline
BMC	64	6	31	58	13	38	61
		9.4%	48.4%	90.6%	20.3%	59.4%	95.3%
BMJ	42	4		20	25	5	26
		9.5%		42.9%	59.5%	11.9%	61.9%
Cell Press	28	28	28	1			28
		100%	3.6%	3.6%			100%
Elsevier	70	38	2	26	17		51
		54.3%	2.9%	37.1%	24.3%		72.9%
JAMA Network	13			13	13	13	13
				100%	100%	100%	100%
The Lancet Group	24	2	1	24	24	24	24
		8.3%	4.2%	100%	100%	100%	100%
Nature Research	63	38	16	53	40		59
		60.3%	25.4%	82.5%	63.5%		93.7%
PLOS	9	9		9	9	1	9
		100%		100%	100%	11.1%	100%
Springer	59	49	50	53	54	49	59
		83.1%	84.7%	89.8%	91.5%	83.1%	94.9%
Taylor & Francis	96	6	4	6	10		18
		6.3%	4.2%	5.2%	10.4%		18.8%
Wiley	91	36	21	24	45	22	58
		39.6%	23.1%	23.1%	49.5%	24.2%	63.7%
*Grand*	*559*	216	126	287	250	152	410
		38.6%	22.5%	51.3%	44.7%	27.2%	73.3%

Journals by BMC, Springer, The Lancet Group, and Wiley mention all five of the guidelines in at least some of their journals. Journals by BMJ, Elsevier, Nature, PLOS, and Taylor & Francis mention four guidelines, and journals by Cell Press and JAMA mention three guidelines.

One hundred and forty-nine journals (26.7%) did not mention any reporting guidelines in their IFAs. These are journals published by BMC (3 or 4.7%), BMJ (14 or 33.3%), Elsevier (19 or 27.1%), Nature (3 or 4.8%), Springer (3 or 5.1%), Taylor & Francis (77 or 80.2%), and Wiley (30 or 33.0%).

Because each reporting guideline has several types of documents to which authors are to adhere (e.g., checklist, flow diagram, abstract), IFAs may specify the expectations for how to demonstrate adherence to the guidelines. Journals may require one or more sources of evidence. All five guidelines include a checklist or essential items, but not all publishers or journals require submission of a completed checklist to demonstrate compliance with the specific guideline. Journals may indicate general adherence by simply referring to the guideline, or may provide other forms of demonstration, such as inclusion of guideline adherence in the manuscript abstract (CONSORT), a flow diagram (CONSORT or PRISMA), or a figure (e.g., SPIRIT), or Essential 10 items (ARRIVE). Table 5A–5E summarize the publishers with IFA-mentioned journals and the types of documents that may be used to demonstrate adherence.

**Table 5:** a. Document Types for CONSORT Mentioned in Journal IFAs. **b.** Document Types for PRISMA Mentioned in Journal IFAs. **c.** Document Types for SPIRIT Mentioned in Journal IFAs. **d.** Document Types for CARE Mentioned in IFAs. **e.** Document Types for ARRIVE Mentioned in IFAs.

**5a**					
Publisher	Journals*	Abstract	Checklist	Flow Diagram	Statement
BMC	64	57	36	5	40
BMJ	42	9	15	7	18
Cell Press	28			19	1
Elsevier	70		16	16	25
JAMA Network	13		13	13	13
The Lancet Group	24	24		18	24
Nature Research	63		52	2	38
PLOS	9		9	9	9
Springer	59		52	4	52
Taylor & Francis	96	2	5	4	8
Wiley	91		20	14	43
*Total*	*559*	*92*	*218*	*111*	*271*
**5b**					
Publisher	Journals*	Checklist	Flow Diagram	Statement	
BMC	64	4	5	12	
BMJ	42	15	9	20	
Elsevier	70	5	3	15	
JAMA Network	13	13	13	13	
The Lancet Group	24	1		24	
Nature Research	63	21	1	21	
PLOS	9	9	9	9	
Springer	59	51		52	
Taylor & Francis	96	4	3	8	
Wiley	91	16	5	43	
*Total*	*531*	*139*	*48*	*217*	
**5c**					
Publisher	Journals*	Checklist	Flow Diagram	Statement	
BMC	64	30	30	36	
BMJ	42	5	3	3	
JAMA Network	13			13	
The Lancet Group	24			24	
PLOS	9	1	1	1	
Springer	59	49		49	
Wiley	91	1	1	22	
*Total*	*302*	*86*	*35*	*148*	
**5d**					
Publisher	Journals*	Checklist	Statement		
BMC	64	26	30		
Cell Press	28		1		
Elsevier	70	1	2		
The Lancet Group	24	1	1		
Nature Research	63		16		
Springer	59	49	50		
Taylor & Francis	96	1	4		
Wiley	91	3	21		
*Total*	*495*	*81*	*125*		
*Total*	*546*	*66*	*214*		
**5e**					
Publisher	Journals*	Essential 10	Guidelines		
BMC	64	2	5		
BMJ	42	1	4		
Cell Press	28		28		
Elsevier	70	2	38		
The Lancet Group	24	2	2		
Nature Research	63	2	38		
PLOS	9	3	9		
Springer	59	49	49		
Taylor & Francis	96	2	6		
Wiley	91	3	35		
*Total*	*546*	*66*	*214*		

* Journal totals reflect only those publishers with at least one IFA-mentioned journal for the specified reporting guideline

#### Journals that published reporting guidelines and their IFAs (Q4)

Seventeen journals published five reporting guidelines (Table 2). The four journals that published ARRIVE all mentioned the guideline in their IFAs. For the six journals that published CONSORT, five recommended or required at least one of the four reporting documents (83%). For the five journals that published PRISMA, four required both a checklist and the flow diagram (80%). The Journal of Clinical Epidemiology (Elsevier), which published CONSORT, PRISMA, and SPIRIT, in its For Authors instructions, listed PRISMA and CONSORT, plus “The appropriate reporting guidance must be cited in the Methods section. Adherence to these checklists will be verified for these methods. Completed checklists must be submitted using file type “S1 File” and will be published alongside accepted articles. For other methods, authors are required to review the EQUATOR Network to identify standards appropriate to their methods, or to state that such standards do not exist.” [47] For SPIRIT, three of the journals (75%) mentioned it in their IFAs, except for Systematic Reviews (BMC) which did not mention it in its IFA (although it mentioned PRISMA). However, only one of the five published journals (20%) mentioned CARE in its IFA, the Journal of Medical Case Reports (BMC). BMJ Case Reports, Headache, Journal of Clinical Epidemiology, and Journal of Dietary Supplements published CARE guidelines, but none mentioned CARE in their IFAs. Systematic Reviews published the SPIRIT guidelines but did not mention SPIRIT in its IFA.

#### Endorsements on guidelines’ websites and in IFAs (Q5)

Of the 559 journals in our dataset, 177 journals are endorsement-listed on one or more of the four reporting guidelines’ websites. We checked if the endorsement-listed journals instructed their authors about the reporting guidelines. A journal that follows through with the web endorsement by instructing authors in submissions is both web-endorsed and IFA-mentioned. The overlaps for each of the four guidelines are as follows:

- For ARRIVE, 76 or 61.3% of the 124 web endorsed journals follow through with IFA mentions
- For CONSORT, 73 or 81.1% of the 90 web endorsed journals follow through with IFA mentions
- For PRISMA, 10 or 21.3% of 47 web endorsed journals follow through with IFA mentions
- For SPIRIT, 39 or 19.6% of the 51 web endorsed journals follow through with IFA mentions

With further analysis of the IFAs of the journals that are listed on the four guidelines’ websites as endorsers (Table 6), we found that journals by The Lancet Group and PLOS instructed their authors on the guidelines they endorsed on the websites (100%). JAMA Network endorsed three guidelines; for the journals that web- endorsed CONSORT and SPIRIT, they also instructed authors to follow these guidelines in their IFAs. For Cell Press, all journals that web-endorsed ARRIVE also mentioned it in their IFAs.

**Table 6:** Do website endorsed guidelines mentioned in journals IFAs?

Publisher		ARRIVE	CONSORT	PRISMA	SPIRIT
BMC	IFA-mentioned	5	35	4	24
	Web-endorsed	41	36	40	34
	Percentage	12.2%	97.2%	10.0%	70.6%
BMJ	IFA-mentioned	1	7	2	1
	Web-endorsed	3	11	3	2
	Percentage	33.3%	63.6%	66.7%	50.0%
Cell Press	IFA-mentioned	15			
	Web-endorsed	15			
	Percentage	100%			
Elsevier	IFA-mentioned	23	9	1	
	Web-endorsed	24	13	1	
	Percentage	95.8%	69.2%	100%	
JAMA Network	IFA-mentioned	0	5		12
	Web-endorsed	1	5		12
	Percentage	0%	100%		100%
The Lancet Group	IFA-mentioned	1	4	1	1
	Web-endorsed	1	4	1	1
	Percentage	100%	100%	100%	100%
Nature	IFA-mentioned	19	1		
	Web-endorsed	21	2		
	Percentage	90.5%	50.0%		
PLOS	IFA-mentioned	7	3	2	1
	Web-endorsed	7	3	2	1
	Percentage	100%	100%	100%	100%
Springer	IFA-mentioned	1	1		
	Web-endorsed	2	1		
	Percentage	50.0%	100%		
Taylor & Francis	IFA-mentioned	0	2		
	Web-endorsed	1	6		
	Percentage	0%	33.3%		
Wiley	IFA-mentioned	4	6		0
	Web-endorsed	8	9		1
	Percentage	50.0%	66.7%		0%

#### Enforcement of reporting guidelines: Peer review reports (Q6)

**Fig 1:**
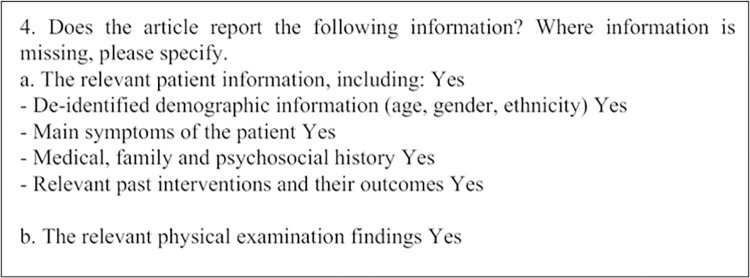
Example of questions for review report by journal of medical case reports.

Although a systematic analysis of enforcement of endorsed reporting guidelines is beyond this study’s scope, it is necessary to conduct a cursory observation to inform further studies. Analyzing the peer review reports from OPR journals, we examined if there was evidence that reviewers checked the adherence to the endorsed reporting guidelines based on the text of their reviews. Table 7 summarizes the OPR journals that published the guidelines and appeared on the lists of the reporting guidelines’ endorsing journals and the journals mentioning the guidelines in their IFAs. The comments from the peer review reports indicate that some reviewers are checking if the authors adhere to the specific reporting guidelines. It should be noted that there are differences for some of the journals between the guidelines they have endorsed and those they have mentioned in their IFAs. It is worth mentioning that the Journal of Medical Case Reports, published by BMC, provides guidance in submitting review reports through questions that include some items from the CARE checklist. For example, reviewers were asked to address specific questions such as in Fig 1.

**Table 7:** Checking adherence to reporting guidelines by peer reviewers.

Journal	Web-Endorsed	Published	IFA-Mentioned	# of Reports Mentioning a Guideline (%)
BMC Medicine # of Articles: 115 # of Reports: 331	ARRIVE, CONSORT, PRISMA	CONSORT	CONSORT, PRISMA, SPIRIT	CONSORT: 4 (1.2%) PRISMA: 15 (4.5%)
	e.g., “CONSORT flow chart is acceptable.” [doi: s12916-020-01851-z] “I advise the authors to include the CONSORT reporting standards [doi; s12916-021-02053-x] “… consider employing the PRISMA” [doi: s12916-020-01623-9] “… authors followed the PRISMA statement” [doi: s12916-021-02161-w]				
PLOS Medicine # of Articles: 74 # of Reports: 326	ARRIVE, CONSORT, PRISMA	CONSORT, PRISMA	ARRIVE, CONSORT, PRISMA	CONSORT: 20 (6.1%) PRISMA:7 (2.1%)
	e.g., “The overall study conforms to CONSORT guidelines for a randomized trial,. . .…” [doi: 10.1371/journal.pmed.1003875] “Please complete a checklist for the most appropriate reporting guideline, e.g., PRISMA (we suggest PRISMA 2020. https://journals.plos.org/ …) [doi: 10.1371/journal.pmed.1003861]				
Trials (BMC) # of Articles: 399 # of Reports: 728	CONSORT, PRISMA, SPIRIT	CONSORT, SPIRIT	CONSORT, SPIRIT	CONSORT: 14 (1.9%) PRISMA: 5 (0.6%) SPIRIT: 328 (45.1%)
	e.g., “The study appears to have been rigorously conducted and the enclosed CONSORT checklist is complete and shows where the necessary information is given in the paper.” [doi: 10.1186/s13063-021-05742-3] “See explanation of Item 8b on CONSORT checklist.” [doi: 10.1186/s13063-020-04850-w “There is no indication, however, of the number of studies included through author knowledge in PRISMA diagram/results” [doi: 10.1186/s13063-022-06000-w] “Thank you for submitting a completed detailed SPIRIT checklist for your RCT.” [doi: 10.1186/s13063-020-04904-z] “I am reviewing this form the perspective of SPIRIT and I have structured my comments accordingly.” [doi: 10.1186/s13063-022-06168-1]				
PLOS Biology # of Articles: 83 # of Reports: 253	ARRIVE	ARRIVE	ARRIVE, CONSORT, PRISMA	PRISMA: 2 (0.8%)
	e.g., “Full PRISMA flowchart would help …. . . at least like to see a PRISMA diagram showing the results of screening processes.” [doi: 10.1371/journal.pbio.3001511] “. . .. . . in providing more transparency … a PRISMA flow chart may have much more intuitive and easy to follow …. . .” [doi: 10.1371/journal.pbio.3001556]				
Journal of Medical Case Reports # of Articles: 95 # of Reports: 225		CARE	CARE, CONSORT	CARE: 1 (0.4%)
	“Background needs much work. It is too concise. You could consult CARE guidelines for case report preparation. You should briefly summarize in 2 paragraphs why this case is unique and include medical literature references.”[doi: 13256_2022_3642_ReviewerReport_V0_R1]				

Questions in Fig 1 correspond quite closely to items on the CARE checklist:

“5. Patient Information:

- De-identified patient specific information.
- Primary concerns and symptoms of the patient.
- Medical, family, and psycho-social history including relevant genetic information.
- Relevant past interventions with outcomes.

6. Clinical Findings–Describe significant physical examination (PE) and important clinical findings.

……” [46, Checklist]

Journal of Medical Case Reports has Protocol Editors who check and mention items defined by CARE. PLOS Biology, PLOS Medicine and BMC Medicine provided no specific questions to peer reviewers corresponding to reporting guidelines.

## Discussion

For the journals that published specific reporting guidelines (Q1), only the four journals that published ARRIVE also mention it in their IFAs. For the 13 journals that published the four guidelines, not all mention them in their IFAs (as few as 20%). There may be reasons that these journals have not instructed authors to adhere to the guidelines the journals published. Although it is important for a journal to publish the guidelines, the endorsement of a guideline should also be reflected in IFAs because when authors prepare manuscripts, they are more likely to check IFAs to be informed about which guidelines to follow.

The analysis of journals that are listed as endorsers on the websites of the reporting guidelines (Q2) reveals wide variation in how publishers and journals endorse reporting guidelines. The inclusion of journals on at least one guideline’s website ranged from 5.1% for Springer journals to 92.3% for JAMA journals (Table 3). Clearly, the types of studies journals publish will influence which guidelines are appropriate for endorsement. Still, prominent guidelines such as ARRIVE and CONSORT were only endorsed by 22.2% and 15.9% of the journals, respectively. Overall, only 31.7% of journals (177 of 559) endorsed at least one of the four prominent guidelines.

The prevalence of journal endorsement of reporting guidelines, as reported by the guidelines themselves, is in contrast to the inclusion of guidelines in journal IFAs where there is greater representation (Q3). Four hundred and ten of the 559 journals (73.3%) include reference to at least one of the five reporting guidelines in their IFAs, with four publishers having all of their journals include mention of at least one guideline. CONSORT, which addresses reporting of randomized trials, is most prevalent, being mentioned in more than half of the journals examined. Shamseer et al. [45] also examined IFAs requirements for specific documents of CONSORT and found that submissions of trial manuscripts require a checklist (38%) and flow diagram (39%). They conclude: “… specific instructions on how CONSORT should be used by authors are inconsistent across journals and publishers. Publishers and journals should encourage authors to use CONSORT, where relevant, and set clear expectations for authors about compliance with CONSORT.” (Abstract)

Individual journals may provide multiple ways for authors to demonstrate guideline adherence. The different methods publishers use to demonstrate adherence to reporting guidelines and their frequency by journals also indicate that there is no universally adopted way for adherence to be demonstrated. For CONSORT, flow diagrams and statements were used by all publishers to varying degrees, although checklists were more common for some publishers. Statements were also more prevalent for publishers for SPIRIT, PRISMA and CARE adherence. This may be confusing for both authors and reviewers where there is a lack of consistency within and across journals.

For the 17 journals that published reporting guidelines (Q1), there was greater consistency in how they instructed authors in their IFAs to adhere to the guidelines (Q4), but not all the journals did so for all of the guidelines they published. One would assume that if a journal published a guideline, they would also instruct authors to adhere to the guideline by mentioning it in their IFA.

As with Q3 above, there was a lack of consistency in how journals endorsed guidelines and how they included them in their IFAs (Q5). This can also result in confusion for authors when specific guidelines are not mentioned nor is a link to a specific reporting guideline included. However, authors who are exploring publication venues may find reporting guidelines from the editorial policy made available by the publisher elsewhere. Taylor and Francis, which simply lists 17 reporting guidelines (that is, five links from a journal’s IFA to reporting guidelines’ page may not provide enough guidance to authors on which guidelines should be used for specific journals. Without specific guidance from journals, authors may self-select which guidelines to follow, believing that the guideline usage is optional. These concerns are supported by the findings of the study of a random sample of 200 articles [48]. The researchers reported on the use of four major reporting guidelines and found that the correct use of the guidelines varied for articles in different studies: CONSORT (64%), PRISMA (22%), ARRIVE (14%), and another guide we did not study, CHEERS for economic evaluation (42%).

The open peer review reports examined (Q6) reveal that some reviewers do check if the authors used a reporting guideline correctly or suggest the specific documents of the guidelines. Based on the sample of articles and associated reviews examined, the percentage of reviewers who mention adherence to reporting guidelines was under 10% for all guidelines, except for the BMC journal Trials, where mention of SPIRIT guidelines by reviewers was 45.1%. It should be noted that not all mentions of SPIRIT were reviewer-initiated. For example, 116 review reports included the statement “This is a review by the SPIRIT protocol editor for Trials.” Because protocol editors reviewed manuscripts for SPIRIT adherence.

There is disagreement as to whether peer reviewers should be involved in checking reporting guidelines [33, 35, 49]. The helpfulness of guidelines for reviewers in reviewing the completeness of reviews was studied in two randomized trials with the conclusion that “it was not useful to implement the tested intervention to increase reporting completeness in published articles. Other interventions should be assessed and considered in the future.” [33, p 2/3] One interpretation for this finding could be that it is late in the research process for authors to have to revisit their work if found not to adhere to expected guidelines at the review stage. However, the reviewers can confirm if authors have followed guidelines. A survey of authors, reviewers and editors [28] showed that reviewers, more than editors, were willing to check and enforce appropriate reporting guidelines.

An important feature of guideline adherence is to ensure that studies are replicable. Although we do not believe this falls solely on the shoulders of the reviewers to ensure replicability of the study, many guidelines highlight this important aspect of research by requiring sufficient detail of the data, their collection and analysis.

There are limitations to the current study. Although we have focused on prominent publishers that are responsible for many influential journals, we recognize that there are many publishers of biomedical and health-related research who may approach the endorsement of reporting guidelines differently. Sampling was purposive and not randomized, therefore, we cannot generalize the findings to all biomedical publication venues. Furthermore, we have focused on five of the more commonly used reporting guidelines. Variations of these guidelines exist, and many more guidelines were not included in the study. Although earlier research has found that that stricter adherence to reporting guidelines will improve the quality of the reports [18, 21, 23], the direct influence of the guidelines on the quality of the published research requires further investigation involving editors and other stakeholders.

## Conclusions

This study has revealed how five major guidelines, representing five common biomedical study types in biomedical fields, have been adopted by journals from 11 prominent publishers of biomedical and health-related journals. The endorsement of these guidelines varies, depending on the nature of the journals. CONSORT and PRISMA, representing randomized trials and systematic review studies, respectively, are the most widely endorsed guidelines; ARRIVE also has the largest endorsers list and is mentioned most by journals that publish research involving animals.

Currently, there is no universal endorsement of reporting guidelines by biomedical publishers nor ways to demonstrate adherence to the endorsed reporting guidelines. There is also a lack of agreement on what constitutes an endorsement; finer nuances for the types of endorsements are needed.

To increase the use of endorsed reporting guidelines across journals, we recommend: 1) explicit instructions on endorsed guidelines be provided for authors (i.e., require, must); 2) publishers should implement tools for journals to ensure that the guideline adherence expectations are met; 3) authors, the most important entity in scientific publishing, need to be aware of the relevant reporting guidelines and the tools (e.g., the reporting checklists for medical researchers [50]; 4) reporting guidelines should update their endorsers with additional fields including the publisher and endorsement date.

Future research should investigate the explicitness of IFAs and the effect of requirements on the correct use of the guidelines. Further research is needed to study the experiences of authors and peer reviewers of the journals that endorsed reporting guidelines. Studies also need to explore the best practices for enhancing adherence of endorsed guidelines or mandating use of reporting guidelines. Similar studies of other important guidelines are also needed.

## Supporting information

S1 File (ZIP)
